# Embodied innovation and regulation of medical technoscience: transformations in cancer patienthood

**DOI:** 10.1080/17579961.2015.1106103

**Published:** 2016-02-02

**Authors:** Anne Kerr, Sarah Cunningham-Burley

**Affiliations:** ^a^ School of Sociology and Social Policy, University of Leeds, England, UK; ^b^ School of Molecular, Genetic and Population Health Sciences, Centre for Population Health Sciences and Centre for Research on Families and Relationships, University of Edinburgh, Scotland, UK

**Keywords:** Cancer, genomics, patients, participation, work

## Abstract

Biomedical advances are transforming the diagnosis and treatment of disease. Patienthood is also transforming, as patients actively participate in research, innovation and regulation of novel technologies and therapies. In this paper we explore the new kinds of practices that patients are performing in their roles as research subject, co-researchers, donors, campaigners, representatives and consumers of novel stratified therapies. We outline their embodied contributions to clinical trials, biobanks and stratified therapies prior to, during and after having cancer. Exploring how patienthood involves donating more than tissue or data to these developments, we consider their emotional and identity work which informs and shapes the novel diagnostics and therapies being developed. We also consider how this kind of work is stratified according to the social and biological location of participants, and end by reflecting on the implications of our analysis for the organisation and regulation of biomedicine.

## 1. Introduction

Across the twentieth century and into the current century we have seen widespread advances in our abilities to prevent, detect and treat a range of diseases. Rapid developments in the so-called ‘post-genomic’ research and medicine also offer the possibility of much more targeted interventions to prevent and tackle diseases, turning conditions like cancers into chronic rather than terminal conditions. These developments in medical science and technologies are celebrated in popular and policy narratives because of their potential to improve our lives and ensuring an effective and efficient health care system. Yet this agenda comes with risks and questions attached. Most notably, extensive research, monitoring and interventions in the interests of conquering disease has the potential to make patients of us all as we attend to the possibility of developing or lapsing into disease through the lifecourse.^1^ 1 Carlos Novas and Nikolas Rose, ‘Genetic Risk and the Birth of the Somatic Individual' (2000) 29 *Economy and Society* 485; Adele E Clarke, ‘Biomedicalization' in William Cockerham, Robert Dingwall and Stella R. Quah (eds), *The Wiley Blackwell Encyclopedia of Health, Illness, Behavior, and Society* (Wiley-Blackwell, 2014); S Scott, L Prior, F Wood and J Gray, ‘Repositioning the Patient: The Implications of Being “At Risk”' (2005) 60 *Social Science & Medicine* 1869.


Being a patient in the contemporary post-genomic era is freighted with moral and political decisions, social practices and new organisational forms. This raises questions about the ways in which biomedical research, diagnosing, monitoring and treatment of disease or its possibilities are changing patienthood, bringing new demands for particular kinds of emotional and embodied practices on the part of patients or patients-in-waiting.^2^ 2 Kirsten Bell, ‘Biomarkers, the Molecular Gaze and the Transformation of Cancer Survivorship' (2013) 8 *Biosocieties* 124; Eric J Cassell, *The Nature of Suffering and the Goals of Medicine* (Oxford University Press, 2nd edn 2004); Kyra Landzelius, ‘Introduction: Patient Organization Movements and New Metamorphoses in Patienthood' (2006) 62 *Social Science & Medicine* 529; Nicky James, ‘Emotional Labor: Skill and Work in the Social Regulation of Feelings’ (1989) 37 *Sociological Review* 15. As sociologists of biomedicine have argued, we need to think carefully about how these practices are distributed, experienced and valued, and the ways in which this is reconfiguring the implicit contract between the individual and the healthcare system.^3^ 3 Catherine Waldby, ‘Stem Cells, Tissue Cultures and the Production of Biovalue' (2002) 6 *Health* 305; Carlos Novas, ‘The Political Economy of Hope: Patients’ Organizations, Science and Biovalue' (2006) 1 *Biosocieties* 289; Nikolas Rose and Carlos Novas, ‘Biological Citizenship' in Aihwa Ong and Stephen J Collier (eds), *Global Assemblages: Technology, Politics, and Ethics as Anthropological Problems* (Blackwell, 2005); Catherine M Will, ‘Mutual Benefit, Added Value? Doing Research in the National Health Service' (2011) 4 *Journal of Cultural Economy* 11.


This means engaging with the interactions which make up the experiences and notions of contemporary patients, not just amongst and between patients and doctors, but across the innovation spectrum, including more hidden processes of patient involvement and representation in biobanking, trials and data analytics and cultural industries of social media and patient advocacy.^4^ 4 Aline Sarradon-Eck, Juliette Sakoyan, Alice Desclaux et al, ‘“They Should Take Time”: Disclosure of Clinical Trial Results as Part of a Social Relationship' (2012) 75 *Social Science & Medicine* 873; Richard Tutton and Barbara Prainsack, ‘Enterprising or Altruistic Selves? Making up Research Subjects in Genetics Research' (2011) 33 *Sociology of Health & Illness* 1081; Richard Tutton, ‘Personalizing Medicine: Futures Present and Past' (2012) 75 *Social Science & Medicine* 1721; Effy Vayena and Barbara Prainsack, ‘Regulating Genomics: Time for a Broader Vision' (2013) 5 *Science Translational Medicine* 1. To explore this we need to approach patienthood as dynamic, multi-faceted and performative – as woven from a web of socio-technical processes rather than fixed in individual experiences or attributes.^5^ 5 Adele E Clarke, Janet K Shim, Laura Mamo et al, ‘Biomedicalization: Technoscientific Transformations of Health, Illness, and US Biomedicine' (2003) 68 *American Sociological Review* 161. We need to understand these processes, not by normative argument about whether one would or should uptake new technology, but by considering how bodies, emotions and subjectivity feature in a wider set of decisions and discussions about efficacy, use and entitlements to biomedical technoscience.^6^ 6 Gayle A Sulik, ‘Managing Biomedical Uncertainty: The Technoscientific Illness Identity' (2009) 31 *Sociology of Health & Illness* 1059.


Although there is clearly a need to consider the embodied subjectivities of all of the different actors who encounter biomedicine – scientists, healthcare practitioners, policymakers – here we turn our attention to patients’ bodies, given that these are principal bodies on which technoscience impinges and indeed develops. We focus on how their bodies and emotions co-produce the innovation and regulation of biomedical technoscience.^7^ 7 Steven Epstein, *Impure Science: AIDS, Activism, and the Politics of Knowledge* (University of California Press, 1996); J Francisca Caron-Flinterman, Jacqueline EW Broerse and Joske FG Bunders, ‘Patient Partnership in Decision-Making on Biomedical Research: Changing the Network' (2007) 32 *Science Technology & Human Values* 339; Michel Callon and Vololona Rabeharisoa, ‘The Growing Engagement of Emergent Concerned Groups in Political and Economic Life: Lessons from the French Association of Neuromuscular Disease Patients’ (2008) 33 *Science Technology & Human Values* 230. If patients are, like philosophers, seeking particular answers to their condition, and the human condition besides, how is this achieved via their bodies and technologies combined? And what of the patients who do not participate or are not acknowledged in these processes? What is their embodied experience and relationship to biomedical innovation and regulation; where does the resource offered by their body generate value; and who benefits? As this mention of value reminds us, patients are increasingly asked to participate in biomedical innovation as research subjects, evaluators, monitors, campaigners, commentators, donors and fund-raisers. Despite the fact that these practices generate value, they are not often considered or remunerated as ‘work’. There is also little reflection on what kinds of patients are able and willing to contribute in these ways.^8^ 8 For an important recent contribution on the work of donation and research subjects by non-patients, see Melinda Cooper and Catherine Waldby, *Clinical Labor: Tissue Donors and Research Subjects in the Global Bioeconomy* (Duke University Press, 2014).


To explore these questions we focus our attention on cancer in the post-genomics era. We have chosen cancer because it is both ubiquitous and the site of rapid biomedical advancement. It is estimated that more than one in three people will be diagnosed with cancer over the course of their lives,^9^ 9 PD Sasieni, J Shelton, N Ormiston-Smith et al, ‘What is the Lifetime Risk of Developing Cancer?: The Effect of Adjusting for Multiple Primaries’ (2011) 105 *British Journal of Cancer* 460. and so almost everyone is touched by cancer as family members, friends, charitable donors and citizens. Nevertheless, these experiences vary dramatically as the stubborn deprivation gap in the incidence and prognosis of cancer attests.^10^ 10 B Rachet, LM Woods, E Mitry et al, ‘Cancer Survival in England and Wales at the End of the 20th Century' (2008) 99 *British Journal of Cancer* S2; J Ferlay, I Soerjomataram, M Ervik et al, ‘GLOBOCAN 2012 v1.0, Cancer Incidence and Mortality Worldwide: IARC CancerBase No 11' (*International Agency for Research on Cancer,* 2013) http://globocan.iarc.fr (accessed 26 March 2015). Inflected through the politics of gender, class, age and ethnicity, experiences of cancers vary dramatically. At the same time rapid developments in cancer genomics have introduced new clinical trials, treatment regimes and monitoring practices.^11^ 11 Eric T Juengst, Michael A Flatt and Richard A Settersten Jr, ‘Personalized Genomic Medicine and the Rhetoric of Empowerment' (2012) 42 *Hastings Center Report* 42 (5): 34–40; Alberto Ocana and Atanasio Pandiella, ‘Personalized Therapies in the Cancer “Omics” Era' (2010) 9 *Molecular Cancer*.


There is also a veritable industry of charitable donations, research initiatives, treatment centres and cultural products associated with cancer where patients are consumers and producers of the disease, diagnosis and therapy. Cancer is at the frontier of novel ways of ‘doing medicine and health care’, embracing Prediction, Prevention, Personalisation and Participation, or the ‘P4 paradigm’ as it is known in biomedicine.^12^ 12 Leroy Hood and Mauricio Flores, ‘A Personal View on Systems Medicine and the Emergence of Proactive P4 Medicine: Predictive, Preventive, Personalized and Participatory' (2012) 29 *New Biotechnology* 613. Patients' bodies are involved in myriad ways with contemporary cancer research, innovation, care and regulation. In what follows we explore three key sites of embodied biomedical innovation and regulation: (i) cancer drugs trials (ii) national genomics data initiatives and (iii) public funding for targeted treatments, in order to think more broadly about the ways (particular kinds of) patients bodies are implicated in innovation and regulation in the post-genomics era.

## 2. Being on trial

Participation in clinical trials is now an established part of cancer therapy for growing numbers of patients, not just those at an advanced stage of the disease.^13^ 13 Peter Keating and Alberto Cambrosio, *Cancer on Trial: Oncology as a New Style of Practice* (University of Chicago Press, 2012); Sarradon-Eck et al (n 4); Vinh-Kim Nguyen, ‘Antiretroviral Globalism, Biopolitics, and Therapeutic Citizenship' in Aihwa Ong and Stephen J Collier (eds), *Global Assemblages: Technology, Politics, and Ethics as Anthropological Problems* (Blackwell, 2008); Tiago Moreira and Paolo Palladino, ‘“Population Laboratories” or “Laboratory Populations”? Making Sense of the Baltimore Longitudinal Study of Aging, 1965–1987' (2011) 42 *Studies in History and Philosophy of Biological and Biomedical Sciences* 317. This is reflected in the practices of large national organisations like Cancer Research UK, who provide ‘Find a Trial’ services online to give patients the chance to access new therapies. As drugs have developed to target particular configurations of cancer genomics, patients can search according to their cancer subtype, as well as the drug the trial involves. Patients groups are also actively involved in campaigning to increase access to trials amongst their constituencies, working in partnership with trial providers across the private and public sectors.^14^ 14 Aaron Panofsky, ‘Generating Sociability to Drive Science: Patient Advocacy Organizations and Genetics Research' (2011) 41 *Social Studies of Science* 31. As Moreira and Wills have argued, debates about design, organisation, and evaluation extend beyond the clinical community involving patients in new ways, as consumers and producers of trials and their results.^15^ 15 Catherine Will and Tiago Moreira (eds), *Medical Proofs, Social Experiments: Clinical Trials in Shifting Contexts* (Ashgate, 2010).


However, this proliferation of trials rests on an uneasy tension around the public and personal benefits of trial participation.^16^ 16 Oonagh Corrigan, ‘Empty Ethics: The Problem with Informed Consent' (2003) 25 *Sociology of Health & Illness* 768. The scientific logic of later phase trials is that participants are assigned randomly to the new experimental treatment or the ‘control’ arm of the trial which uses conventional treatment, in order that the safety and efficacy of the new drug can be established; as a result new trial designs have to embrace molecular stratification through ever more complex protocols. But it is widely recognised that patients often join these trials because they want to access the experimental treatment, in the hope that it will prove to be more effective than conventional treatment.^17^ 17 Christopher Daugherty, Mark J Ratain, Eugene Grochowski et al, ‘Perceptions of Cancer Patients and their Physicians Involved in Phase I Trials’ (1995) 13 *Journal of Clinical Oncology* 1062; Katie Featherstone and Jenny L Donovan, ‘“Why Don't They Just Tell Me Straight, Why Allocate It?”: The Struggle to Make Sense of Participating in a Randomised Controlled Trial' (2002) 55 *Social Science & Medicine* 709; Mary Dixon-Woods and Carolyn Tarrant, ‘Why Do People Cooperate with Medical Research? Findings from Three Studies’ (2009) 68 *Social Science & Medicine* 2215. Participation in trials has, for many professionals and patients, become an established part of contemporary cancer care. Their proliferation means that cancer professionals, whether in developed or developing countries, work across research and clinical practice, negotiating and reproducing tensions around altruistic and personal benefits when engaging with patients taking part in trials. As Catherine Will has found in ethnographic research, professionals’ ‘formal and informal strategies for living with this distinction may have the unintended consequence of making research appear supplementary to rather than simply different from clinical care'.^18^ 18 See Will (n 3) 11.


This involves patients in devoting time and energy to involving themselves as trial participants – providing information and updates, travelling to trial sites and interviews and keeping a close eye on their health and any side-effects from treatment. This situation is further complicated by the targeting of trials and treatment; in order to achieve the required number of participants, trials may increasingly require patients to travel. Patients make this kind of investment in travel and experimental treatment not simply because of a sense of altruism but because of a desire to access treatments not available locally. In countries where publically funded healthcare is not widely available, participating in commercially organised trials can also be a route to conventional treatments and better basic healthcare. As Petryna notes, there is a wide diversity of investments and experiences of trials amongst patients, depending on their social location and the progression of their illness. For those who are very ill or impoverished, clinical trials might present ‘their best medical option rather than … “mere” experimentation’, and for others trials might also be a source of income.^19^ 19 Adriana Petryna, *When Experiments Travel: Clinical Trials and the Global Search for Human Subjects* (Princeton University Press, 2009) 2–3. However, there have been ethical dilemmas regarding participation in cancer screening trials, the control arms of which do not necessarily give access to standard treatments available in other countries but not typically in the location of the trial.^20^ 20 Eric J Suba, ‘US-funded Measurements of Cervical Cancer Death Rates in India: Scientific and Ethical Concerns’ (2014) 11 *Indian Journal of Medical Ethics* 167.


Despite this proliferation of trials, there is little qualitative information on patients’ experiences of participation or non-participation in novel cancer trials, particularly the kinds of embodied, emotional practices this involves for some (but not all) patients. Most of the data available are based on quantitative measures of quality of life conducted or participant experiences as part of the trial evaluations and piloting to improve trial design and operating procedures.^21^ 21 Nicola Mills, Jane M Blazeby, Freddie C Hamdy et al, ‘Training Recruiters to Randomized Trials to Facilitate Recruitment and Informed Consent by Exploring Patients’ Treatment Preferences’ (2014) 15 *Trials* 323. There are, however, a number of patient testimonies online that give some insights into what it is like to be ‘on trial’, albeit typically from a privileged, largely middle-class, perspective. These narratives are performative,^22^ 22 Judith Butler, ‘Performative Acts and Gender Constitution: An Essay in Phenomenology and Feminist Theory' (1988) 40 *Theatre Journal* 519; Judith Butler, *Undoing Gender* (Routledge, 2004). in that they constitute part of the work patients do in participating in trials. Such stories about trial participation typically form part of a clinical trial organisation's website and comprise one way in which people can be encouraged to join clinical trials; they also create the hybrid identity of patient/research subject. For example, in the Stanford Cancer Institute blog,^23^ 23 Patient Stories Patients share their experiences with participating in clinical trials. Stanford Medicine Cancer Institute Stanford CA, http://med.stanford.edu/cancer/trials/patient-stories.html (accessed 5 November 2015). patients give testimony about the value and benefits of being in a trial, describing their emotional journey from diagnosis through treatment to survivorship. Trials here are presented as their ‘best option’ and doctors as ‘healers’ who are offering patients the chance of the most advanced treatments.

In these accounts, survivors emphasise the need to be hopeful and pro-active rather than fearful or passive in the face of cancer. In accounting for the sacrifice of travelling long distances to take part in trials because of the benefits this could bring to their own health and the future of a cure for others, and telling stories of hope, sacrifice and participation, participants are doing emotional work^24^ 24 See James (n 2); Arlie Hochschild, *The Managed Heart: Commercialization of Human Feeling* (University of California Press, 3rd edn 2012). as well as embodied physical work as part of being on trial and producing value in the bioeconomy. Discourses of hope are produced and reproduced through the clinical trials industry and patient narratives about their involvement in novel trials. Research suggests that, in relation to trials generally, patients hope that they are in the treatment arm,^25^ 25 Chiara Catania, Tommaso De Pas, Aron Goldhirsch et al, ‘Participation in Clinical Trials as Viewed by the Patient: Understanding Cultural and Emotional Aspects which Influence Choice' (2008) 74 *Oncology* 177; D Lindström, I Sundberg-Petersson et al, ‘Disappointment and Drop-Out Rate after Being Allocated to Control Group in a Smoking Cessation Trial' (2010) 31 *Contemporary Clinical Trials* 22; Sandra Meinich Petersen, Vibeke Zoffmann, Jesper Kjaergaard et al, ‘Disappointment and Adherence Among Parents of Newborns Allocated to the Control Group: A Qualitative Study of a Randomized Clinical Trial' (2014) 15 *Trials* 126. and that consent to randomisation is not fully informed, raising challenges for patients and clinicians and others engaged in clinical trial recruitment.^26^ 26 See Featherstone and Donovan (n 17); C Behrendt, T Goelz, C Roesler et al, ‘What Do Our Patients Understand About Their Trial Participation? Assessing Patients’ Understanding of Their Informed Consent Consultation About Randomised Clinical Trials’ (2011) 37 *Journal of Medical Ethics* 74. New cancer trials in the era of stratified medicine may reshape discourses of hope embedded in the relationship between patient, trial recruiter and trial organisation and the role of drug treatment.

There are, however, less welcome emotions and bad feelings to be negotiated as part of trial participation too, although these kinds of practices are not well documented. One report by Eli Lilly,^27^ 27 Eli Lilly & Company, ‘Improving the Patient Experience of Clinical Trials’ London, 17 April 2014 www.lilly.co.uk/global/img/UK/Patient/Lilly-PAG-Report-FINAL-WEB-COPY.pdf accessed 6 October 2014. a large trial provider, nevertheless gives some glimpses into this, noting patients’ disappointments and poor experiences with exclusion, information, consent forms, recruitment and the ending of trials. This can include not being able to access the trial at all, the level of medical support they would like during or after the trial, or finding out that they have been on the ‘control’ arm of the trial as opposed to the new treatment, and so have not had any potential advantage from their participation. These experiences highlight the uneasy tensions around patients’ sense of entitlements to participate and benefit from trials, profit and the provision of public benefit by virtue of their participation. This suggests a reconfiguring of relationships between clinical care and research: being a patient, a research participant and a good biological citizen involves emotional work to handle the conflicting sense of responsibilities and rights involved.^28^ 28 See Rose and Novas (n 3).


Attention has also recently turned to the fact that patients’ experience of side effects during their participation in trials are not always taken into account in the reporting and analysis of the trial results, because they can be ‘filtered’ by clinicians in such a way as to downgrade the severity of certain symptoms or even miss them entirely. Initiatives are now underway to address these deficiencies, through patients reporting directly on symptoms as part of the trial design,^29^ 29 Ethan Basch, ‘The Missing Voice of Patients in Drug-Safety Reporting' (2010) 362 *New England Journal of Medicine* 865. for example via digital technologies to enable ‘real-time’ reporting of their symptoms and side-effects: a form of ‘participatory ethnography’ using mobile phones.^30^ 30 Ethan Basch and Amy P Abernethy, ‘Supporting Clinical Practice Decisions With Real-Time Patient-Reported Outcomes’ (2011) 29 *Journal of Clinical Oncology* 954; Clark C Freifeld, Rumi Chunara, Sumiko R Mekaru et al, ‘Participatory Epidemiology: Use of Mobile Phones for Community-Based Health Reporting' (2010) 7(12) *PloS Med* e1000376. As some respondents in the Lilly report noted, however, this brings additional responsibilities to patients to act as data-recorders, which can be demanding, especially if the recording template is not easy to complete.

Patients’ participation in trials is also extending as some of them become more involved in evaluation of trials and efforts to redesign them to improve participation and experiences, with much of this work focusing on how to improve patient understanding of the randomisation process in order to manage expectations of the trial.^31^ 31 JL Donovan, L Brindle and N Mills, ‘Capturing Users’ Experiences of Participating in Cancer Trials’ (2002) 11 *European Journal of Cancer Care* 210. The Lilly report also points to patients’ interests in giving more information to staff about how their lives were affected by being on the trial and in exchanging emotional support with other patients and families, perhaps in the form of a support group. These developments put patients’ emotions to work in new ways, to support themselves and each other via exchange of information.

The expansion of clinical trials beyond the affluent West, particularly for vaccines for cancers such as cervical cancer (the biggest killer of women in the developing world), also brings new but different kinds of participatory practices. These trials give more women access to screening and treatments that are not otherwise available; but, there are many ethical and legal tensions around their funding, design and the processes of obtaining informed consent. There is an industry of commercial organisations involved in a global search for participants in trials.^32^ 32 See Petryna (n 19). The low costs of running trials in developing countries and history of limited access to treatments amongst potential participants makes them attractive to pharmaceutical companies, but there have been controversies and allegations of under-reporting side-effects, and implicit coercion of participants in these trials because of their fears that their access to healthcare for their family would be compromised should they decline to take part. Participating in trials in these contexts transforms patients’ bodies into a resource for the family, and involves the emotional work of managing responsibilities and obligations to kin.^33^ 33 SciDev Net, ‘Ethics left behind as drug trials soar in developing countries’ The Guardian (London, 4 July 2011) www.theguardian.com/global-development/2011/jul/04/ethics-left-behind-drug-trials-developing (accessed 6 October 2014).


Taken together these developments suggest a broadening and deepening of patients’ involvement in designing, considering, participating in, evaluating and recruiting others to clinical trials, drawing on their embodied experiences and emotions when having treatment and managing and recording its effects. As Jespersen and colleagues have noted, clinical trials involve participants in ‘entangled processes of bodywork, where data are extracted, objectified bodies are manipulated and care practices address the emotional, social and mundane aspects of the participants' everyday lives’.^34^ 34 Astrid P Jespersen, Julie Bonnelycke and Hanne H Eriksen, ‘Careful Science? Bodywork and Care Practices in Randomised Clinical Trials’ (2014) 36 *Sociology of Health & Illness* 655. There has also been a growth in the numbers of patients who are involved in trials as a routine part of their treatment and pressures to include more diverse populations in trials to improve applicability of results, which means that these practices are now being performed by more diverse kinds of bodies. However, there are clearly very different kinds of practices by participants depending on their position on the spectrum of affluent, active and articulate patients at once involved in challenging and co-producing trials and their results to deprived, vulnerable and dependent patients who are more likely to be providing their bodies as resources for the trial in exchange for more basic kinds of care for themselves and their families which affluent patients might take for granted. But, taken together, these practices make trials work, enabling corporations and healthcare bodies to develop and market treatments that generate both private and public benefit. Participants in trials leave their biological, social and emotional traces in the data, technologies and discourses they produce, but the work involved in their generation is largely invisible.

## 3. Genomes ‘r’ us

In addition to their participation in clinical trials, patients and populations are increasingly involved in a range of large-scale biobanking initiatives. As a global example of neoliberalist tendencies in health and medical science, such initiatives enjoin novel ‘technologies of the self’, where individuals donate tissue, health and social information to research institutions which then mine the vast corpus of data this generates for associations between particular kinds of diseases and biological/social characteristics.^35^ 35 Oonagh Corrigan and Richard Tutton, *Genetic Databases: Socio-Ethical Issues in the Collection and Use of DNA* (Psychology Press, 2004); Klaus Hoeyer, ‘The Ethics of Research Biobanking: A Critical Review of the Literature' (2008) 25 *Biotechnology and Genetic Engineering Reviews* 429; Herbert Gottweis and Alan Petersen (eds), *Biobanks: Governance in Comparative Perspective* (Routledge, 2008). In the era of high throughput sequencing and multi-national, globalised partnerships across the scientific community and biomedical industries, these initiatives are becoming an important part of national health research infrastructure, as the recent development of the 100,000 Genome Project in the UK demonstrates. This project aims to sequence 100,000 whole genomes from National Health Service (NHS) patients and their families affected by a range of diseases, currently focusing on rare disease, cancer and infectious disease. It was established by the Department of Health, with a remit to bring genomic medicine to the NHS and ‘kick start the development of a UK genomics industry’.^36^ 36 Genomics England, Department of Health www.genomicsengland.co.uk/about-genomics-england/how-we-work (accessed 26 January 2015). Like other post-genomic initiatives, the 100,000 Genome Project brings together national healthcare systems, publically funded laboratories, patients’ organisations and patients’ bodies in order to generate a vast array of biological data, including whole-genome, and social information as a platform for innovation, including by the global pharmaceutical industry. Here too, as with trials, we find a melding of care and research, as participants at once provide samples, data and support for repositories and further commercial developments, and receive more regular contact with health practitioner, health monitoring and feedback as a consequence of their participation.

To understand how participating in these initiatives extends the practices of patients and their families we can turn to explore the processes through which they give their consent, and some of the challenges to existing systems of informed consent that this brings. The complex networks through which these platforms are developed and utilised, particularly the dynamic research agenda and multiplicity of potential users of stored tissues and data over time, has challenged traditional processes of informed consent and led to a range of models which also demand other kinds of embodied work from donors and participants.^37^ 37 Keating and Cambrosio (n 13). One such model, for example, is that of ‘dynamic consent’, whereby participants maintain a connection to the repository and give consent to particular uses of their data over the course of their lives.^38^ 38 Jane Kaye, Edgar A Whitley, David Lund et al, ‘Dynamic Consent: A Patient Interface for Twenty-First Century Research Networks’ (2015) 23 *European Journal of Human Genetics* 141. However, the complexities and challenges of this model have led some to call for a different more collective model of consent where members of the public act, on behalf of donors, as custodians of their data.^39^ 39 Kostas Kampourakis, Effy Vayena, Christina Mitropoulou et al, ‘Key Challenges for Next-Generation Pharmacogenomics’ (2014) 15 *Embo Reports* 472. Custodial responsibilities include the need to be educated and informed about the kinds of data and science being performed and to be able to imagine how others might feel about their data being used in particular ways. As with patients involved in clinical trials, we find a proliferation of patients donating to or overseeing biobanks on behalf of other donors. These patients are being asked to donate time, intellectual and emotional work as well as data and tissues that they have produced through their embodied, social agency. This ‘responsibilisation’^40^ 40 G Burchell, ‘Liberal Government and Techniques of the Self' (1993) 22 *Economy and Society* 267. of patienthood brings their labour directly into the production of scientific knowledge and novel healthcare interventions.

These new responsibilities for patients involved in biobanking sit alongside an intensification of what Hoeyer calls ‘ethics work’ that has grown as part of their infrastructures, which includes obligations for donors as well as custodians to reflect on their relationship to their donated materials in new ways.^41^ 41 Klaus Hoeyer, ‘Size Matters: The Ethical, Legal, and Social Issues Surrounding Large-Scale Genetic Biobank Initiatives’ (2012) 21 *Norsk Epidemiologi* 211. As Knoppers et al have also argued, ‘the data deluge that is already engulfing biostatisticians may soon be repeated for citizens’ as they have to work out what they do and do not want to know as findings from biobanks emerge.^42^ 42 Bartha Maria Knoppers, Ma'n H Zawati and Emily S Kirby, ‘Sampling Populations of Humans Across the World: ELSI Issues’ (2012) 13 *Annual Review of Genomics and Human Genetics* 395. This requires particular kinds of embodied reflection and decision-making on the part of participants who might be asked whether or not they would like to know or they think others would want to know about how their bodies respond to particular drugs, their propensity to develop certain diseases in the future for which there are not necessarily preventative measures to be taken, and their capacity to live with the uncertainties this information might generate, should they opt to receive it. To be in a position to form a view or respond to these types of questions participants must draw on their embodied ‘stock of knowledge’^43^ 43 Alfred Schutz, *A Phenomenology of the Social World* (Heinemann Educational Books 1972). about other healthcare experiences and project their selves into the future to imagine how they might feel about other kinds of uncertainties and risks in their lives. These practices are forms of work on one's self and one's futures, involving emotional and ethical management of identity and belonging for individuals, families and patient communities.

The extent to which donors might take on this kind of work, of course, varies according to the location, size and longevity of the projects and initiatives with which they are associated but there is evidence to suggest these initiatives are becoming longer and larger over time, changing the nature of participants’ involvement. For example, increasing recognition of the complexity and heterogeneity of cancer has prompted the integration of institutional biobanks with a view to providing more comprehensive resources of biological and lifestyle information for translational medicine and targeted therapies and interventions.^44^ 44 R William G Watson, Elaine W Kay and David Smith, ‘Integrating Biobanks: Addressing the Practical and Ethical Issues to Deliver a Valuable Tool for Cancer Research' (2010) 10 *Nature Reviews Cancer* 646. This agglomeration has produced large-scale biobanks where donated tissues and data are shared internationally. This raises questions about what kinds of practices are required to transform and steward patients’ data in these complex initiatives, including how patients’ views are represented in this process, for example via global protocols and procedures required to align these banks for research. Here custodians of biobanks, including some patients or former patients, must grapple with how patients’ and donors’ emotions and values are to be traced and worked upon in these extended research processes.

These questions are particularly important in the context of the obligations and entitlements of national or global citizenship within which biobanks operate. When biobanks are positioned as national assets, donation is associated with the duties of national citizenship. For example, countries like the UK have sought to position their comprehensive NHS as an invaluable network of resource generation, recruiting patients as donors and building up a database which will form part of the growing biomedical sector in the UK and attract investment from pharmaceutical companies who will pay to access the information. Here becoming a donor or a ‘lay representative’ is presented as an extension of the obligations of patienthood – a way for patients to ‘give back’ to the nation and grow the bioeconomy for the benefit of future generations. To donate is to make an emotional and material connection with the NHS, the nation and its future citizens.

Biobanks are also configuring patient advocacy in new ways, forming an important dimension of their efforts to support and progress research into their condition. Many disease-specific biobanks have strong relationships with patient advocates, which are vital in the recruitment of donors, particularly for rare diseases.^45^ 45 See Knoppers et al (n 42). Knoppers and colleagues give the example of the Genetic Alliance BioBank,^46^ 46 
www.biobank.org (accessed 27 March 2015). a network of genetic advocacy groups, which has provided 10,000 samples/records to this biobank. Being this kind of active patient involves work to constitute the biobank initiative and generate benefit from its results – patients are also involved in lobbying large national and international banking initiatives to focus attention on the particular type or subtype of disease the group represents, potentially competing with other kinds of rare or common cancers, and implicitly different social groups according to class, race, gender and age (given that cancer is stratified according to social location as well as genetics). For many advocates these banks are not likely to generate direct, personal health improvements, so their investment is in future health benefits for their fellow sufferers or families in the case of hereditary diseases. Patients involved in these kinds of advocacy organisations are however central to the ethical apparatus of these biobanks, forming a resource not just for samples and data but ethical reflection and perspectives which is now routinely gathered as the banks set up their operational and governance procedures. Patient representatives like these are active parties in the production of consent forms and data confidentiality procedures, especially where there are concerns about information becoming accessible to third parties, like insurance companies, and there is a perceived need to protect donors from potential discrimination.

It is also important to note that patient involvement in establishing, resourcing and overseeing biobanks is patterned according to class and social location, with particularly stark contrasts to be found between the work expected of ‘donors’ in affluent and developing countries.^47^ 47 Margaret Sleeboom-Faulkner (ed), *Human Genetic Biobanks in Asia: Politics of Trust and Scientific Advancement* (Routledge, 2009). The history of biobanking in developing countries is marked by colonial relations, and there continue to be concerns over the exploitation of donors as research ‘objects’ rather than active subjects. This has prompted a range of activist organisations to campaign for consultation with prospective participants about the design of the biobank^48^ 48 Margaret Lock, ‘Interrogating the Human Diversity Genome Project' (1994) 39 *Social Science & Medicine* 603. and brought issues of benefit sharing, with donors and communities, into sharp relief. There have also been disputes over the generation of intellectual property from these banks, particularly for global multinationals who are able to access results and generate treatments whilst the donors remain in countries with poor healthcare provision.

Patient participation in cancer biobanks, then, is not a straightforward matter of donating tissues and data, but of actively negotiating a much wider terrain of embodied participation that articulates a range of entitlements and obligations around citizenship, family and disease-group. This includes patients who are directly involved as donors as well as those recruited to the business of ‘ethics work’ or politics around privacy, ownership, resources and recruitment to the databases. Donation or oversight requires that patients draw from and reflect on their own and their fellow patients' experiences, perspectives and emotional responses to particular developments and opportunities as they evolve, rather than a simple ‘giving over’ of their bodily tissue or personal information. However, not all patients are in the same position to participate in the constitution or oversight of biobanks, or to experience the rewards that this kind of activity might generate, for their identity, personal health or financial security or that of their family/fellow patients in the future. While recognising that the agency of individual patients and their collectives actively shapes their involvement in neoliberal scientific enterprises, such as biobanks, including not to become involved, others may feel or be actively marginalised through these processes. Biobanks rely on patients' emotional donations and reflections beyond the period of their active donation, utilising the affective investments of individuals for the benefits of the nation and the various corporate bodies through which this is to be realised.

## 4. Public funding for targeted treatments

So far we have considered a range of practices associated with being a patient-participant in biomedical research in the post-genomics era, especially the emotional and ethical work associated with managing the hopes, challenges and obligations of involvement during and beyond the period of active donation or participation in trials and what patient representatives do when they negotiate the governance, stewardship, benefit sharing and future possibilities of these kinds of initiatives.^49^ 49 Callon and Rabeharisoa (n 7); Tutton and Prainsack (n 4); Courtney Davis and John Abraham, ‘Desperately Seeking Cancer Drugs: Explaining the Emergence and Outcomes of Accelerated Pharmaceutical Regulation' (2011) 33 *Sociology of Health & Illness* 731. We now turn to consider the demands and emotions associated with accessing novel treatments and therapies in the post-genomic era, and the kinds of practices involved in managing the different kinds of clinical, institutional and policy challenges these can bring.

The post-genomic era has brought with it a panoply of expensive cancer drugs for particular sub-types of common cancers like breast or prostate cancer. These drugs are not universally available because of their high costs and the mixed economy of provision across different jurisdictions. In some countries with healthcare systems based on personal insurance cover, the drugs are available to those on the most comprehensive schemes whilst others with no or only basic insurance will not be covered and are unlikely to be able to fund the treatment themselves. In countries with national health services, such as the NHS in the UK, regulation of access to the drugs is complex. Decision-making about drug funding is subject to intense government scrutiny and comment by a range of organisations and companies, including the drug companies involved in manufacturing and setting the price for these drugs and patient and charitable organisations as well as patient and public commentators across social media. In England until recently, this involved two bodies with different remits – the Cancer Drug Fund and the National Institute for Care and Excellence (NICE). The methodologies used by organisations like NICE to assess whether or not to fund expensive drugs rely on Health Technology Assessment algorithms which attempt to quantify quality of life, for example the Quality Adjusted Life Year (QALY). These processes of assessment often mean that cancer drugs are deemed too expensive.

Organisations like NICE also function on the public stage, with its leading members actively involving themselves in debates about the need for drug companies to price responsibly and for the nation state to make difficult decisions which balance individual patients’ interests with that of the public more generally, given the finite budgets available for healthcare. The Cancer Drug Fund is also a highly political body. Set up in 2010 because cancer was deemed a special case for exceptional funding which fell outside of the NICE remit, it has seen its budgets soar to over £280M, but it is still not able to fund all of the treatments requested and recently a range of drugs were removed from its approved list to prevent a predicted over-spend of £100M. Commentators have suggested that the Fund is in difficulty because it has not been able to use mechanisms to drive down the cost of treatments, in the way that NICE has, and they have queried the extent to which cancer drugs (as opposed to other kinds of treatments for cancer or other serious diseases) should be a special case, given these high costs.^50^ 50 Anon, ‘The Cancer Drugs Fund Benign or malignant? A well-meaning gesture is causing more and more trouble’ The Economist (London, 24 January 2015) http://www.economist.com/news/britain/21640343-well-meaning-gesture-causing-more-and-more-trouble-benign-or-malignant (accessed 5 November 2015). However, a range of critics have condemned the Fund's decision to ‘strike off’ drugs from its approved list, on the basis that the benefits of the drugs to individual patients are not being properly assessed.

The case of trastuzumab emtansine (Kadcyla) illustrates some of the complex practices of patients and former patients which form part of these processes of negotiation. Kadcyla, which costs an estimated £90,000 per patient, reportedly prolongs life by six months longer than standard treatment for women who carry the human epidermal growth factor 2 (HER2) gene mutation, which affects an estimated one in five of women with advanced breast cancer. In a recent ruling, NICE did not approve funding for Kadcyla on the grounds that it was not cost effective. NICE determined that the cost of the drug set by the manufacturer, Roche, far exceeded the financial threshold of affordability of QALY of £20–30k. This has also been true of seven other advanced breast cancer drugs that have been brought forward for approval since 2011.^51^ 51 Caroline White, ‘NICE Confirms Advanced Breast Cancer Drug is Too Expensive for NHS’ (2014) 349 *British Medical Journal* g5078. The Cancer Drug Fund does fund Kadcyla treatment but recently ruled that three other similar drugs would only be available on a limited basis.

Breakthrough Breast Cancer has been among the organisations involved in debating these decisions. In the case of NICE and Kadcyla they focused attention on the responsibility of Roche to price in a reasonable way, as did the NICE Chief Executive Officer, Andrew Dillon.^52^ 52 Anon ‘NICE rejects ‘costly’ cancer treatment for NHS use’ National Health Executive, London, 23 April 2014 www.nationalhealthexecutive.com/Health-Care-News/Page-71/nice-rejects-costly-cancer-treatment-for nhs-use (accessed 30 January 2015). Patients’ experiences also play a prominent role in these discussions, with patients talking about their hopes, anger and disappointments with respect to their condition and its treatment, as well as their wider sense of the obligations and entitlements of citizenship and sickness. Patients, clinicians, support groups, charities and manufacturers variously emphasised the benefits of accessing these drugs in terms which challenged conventional measures of months-left-to-live and focused on the vital time that this gives patients to spend with their families, in relatively good health, for example, ‘giving women back a normality to their lives’ in their final months.

These discussions were particularly emotive when conducted in the national media, for example in the blog posts of journalists like Judith Potts, of the *Daily Telegraph*.^53^ 53 See Judith Potts, ‘Is Nice shirking its duty of care by blocking the Abiraterone prostate cancer therapy?’ The Telegraph London February 3rd, 2012 http://blogs.telegraph.co.uk/news/judithpotts/100134923/is-nice-shirking-its-duty-of-care-by-blocking-the-abiraterone-prostate-cancer-therapy/; Judith Potts, ‘Did Nice listen to cancer patients when they rejected Abiraterone and Kadycla?’ The Telegraph London August 15th, 2014 http://blogs.telegraph.co.uk/news/judithpotts/100283239/did-nice-listen-to-cancer-patients-when-they-rejected-abiraterone-and-kadycla/ (accessed 17 October 2014). Potts’ tagline tells us she was diagnosed with cancer in 2008, and this fact is used as a platform on which to build her interventions in the cancer funding debates. She appeals to the experiences of cancer patients when making her argument that these drugs should be publically available. For example, Potts argues, ‘an extra six months of life may seem very little to NICE but it allows the patient the time to say goodbye, to put affairs in order, or perhaps attend a family event’. Although acknowledging the high cost of the drugs, Potts points out that the manufacturer has invested in development and needs to see a return on this investment to be viable; she criticises NICE for being uncaring and immoral for not giving the public a return on their National Insurance payments by refusing to fund this drug. As Lakdawalla and colleagues have argued, patients in these situations can prefer ‘hopeful gambles to safe bets’ and their involvement in lobbying regulatory processes is, in part, a matter of trying to factor in the value of hope into Health Technology Assessment algorithms.^54^ 54 Darius N Lakdawalla, John A Romley, Yuri Sanchez et al, ‘How Cancer Patients Value Hope And The Implications For Cost-Effectiveness Assessments Of High-Cost Cancer Therapies’ (2012) 31 *Health Affairs* 676.


At the same time, patient groups are actively involved in taking these kinds of arguments to the regulators, translating their and others’ experiences of new expensive treatments into the kinds of evidence of ‘unmet need’ that HTA bodies require. They are working together with governmental bodies, NICE and pharmaceutical companies in a review of the Cancer Drug Fund and its rising costs. Here patients’ representatives are doing political work, drawing on their emotional connections and embodied experiences of cancer to try to evaluate particular drugs, quantifying and justifying treatment benefits as set against conventional treatments, e.g. extra time spent with family and reduced side effects and, at the same time, acting as custodians of the public interest, in the context of an ageing population. In other clinical areas, hope is sustained through active efforts by patients to access experimental or unavailable treatments through ‘medical tourism',^55^ 55 Alan Petersen, Kate Seear and Megan Munsie, ‘Therapeutic Journeys: The Hopeful Travails of Stem Cell Tourists’ (2014) 36 *Sociology of Health & Illness* 670. rather than through political lobbying.

These kinds of contributions also give insight into what is involved in being on these kinds of treatments, when they are available. Decision-making about the provision of drugs also takes place locally in the clinic and the regional group in which it sits, and this can involve patients in active negotiation with their clinician and administrative bodies to present their case for treatment. Patients who are more affluent also have the option of purchasing these drugs directly. Patients with a terminal diagnosis are involved in managing the uncertainties of their condition as well as their and their families’ hopes for a future that may not exist, as Brown and de Graaf have argued.^56^ 56 Patrick Brown and Sabine de Graaf, ‘Considering a Future Which May Not Exist: The Construction of Time and Expectations Amidst Advanced-Stage Cancer' (2013) 15 *Health, Risk & Society* 543. This can include ‘constructing, bracketing off or discounting the value of the future’ as a way of coping with uncertainty. Patients also have to weigh technical information as part of this process, for example risks, toxicity and potential benefits, a process which cannot be performed without emotional as well as cognitive work, including encounters with fear and grief.^57^ 57 Ajay Aggarwal and Richard Sullivan, ‘Affordability of Cancer Care in the United Kingdom: Is It Time to Introduce User Charges?' (2014) 2 *Journal of Cancer Policy* 31. They also have to build alliances and manage emotional connections with care givers as part of their efforts to access particular treatments, again practices that the most affluent and socially adept patients are much better placed to perform than those with less social and cultural capital.

Our argument here is that these kinds of intervention are not simply a matter of patient participation in or lobbying for regulation or treatment decision, but in innovation more broadly. It is through the extended availability of novel drug therapies that companies are able to develop a viable market and collect data on patient responses, enabling them to reconfigure the treatment pathway, and other therapies under development. Patients’ variable responses to novel cancer treatment therapies are a crucial platform for understanding the basic aetiology of cancer,^58^ 58 Keating and Cambrosio (n 13). so these kinds of patient interventions in the politics of treatment are as much a constituent part of the innovation process as the embodied practices and emotional work of patients engaged in clinical trials and biobanking. Patient involvement in public and policy discussion and debates about drug cancer treatment availability are also part of the innovation process given that it is as much a political as a technical pursuit in the seamless web of contemporary technoscience.

As with the case of trials and biobanks, however, we must also recognise that the performance and reach of these kinds of patient practices is stratified according to the cancer and the social location of the patients concerned. Patients’ embodied interventions in innovation and regulation of novel therapies is most obviously valued and appreciated in relation to cancers which are already highly visible on the national public stage, and tied into a wider set of political claims for legitimacy/support such as breast cancer and women's rights or prostate cancer and men's rights: cancers where patients ‘fight’ for treatment and rights simultaneously.^59^ 59 Gayle A Sulik, *Pink Ribbon Blues: How Breast Cancer Culture Undermines Women's Health* (Oxford University Press, 2010). These cancers are also preferred candidates for advocacy because there is no strong moral discourse of individual responsibility for their development, in contrast to the much more stigmatised lung cancer. This increases the resonance of patients’ embodied advocacy for novel treatments, at the same time implicitly de-legitimising claims for other kinds of treatments or other kinds of diseases where individual rights are outweighed by perceived responsibilities. Patients’ capacity to lay claim to entitlement to novel therapies is also mediated by class and ethnicity, with cancers affecting predominantly white and middle-class constituencies dominating claims to expensive targeted treatments. Again, patients’ uneven entanglement in the complexities of biomedical innovation and its effects demands a range of emotional, cognitive, reflective, ethical and physical work.

## 5. Conclusion

In this short review of three key dimensions of contemporary cancer research and treatments we have suggested that being a cancer patient nowadays involves a variety of largely overlooked embodied or emotional work which nevertheless makes a vital contribution to biomedical innovation in the post-genomics era. We have also suggested that patients’ capacity or willingness to engage in these practices is likely to be patterned according to their social location and biological conditions and will vary according to the particularities of the research or regulatory process concerned. Patient participation and partnership with healthcare systems, biomedical research platforms and the pharmaceutical industry is essential for the future success of post-genomic medicine. Yet these developments rest on unexamined requirements of the ‘work’ done by patients.

This suggests the need for further analysis of the different ways in which some patients are contributing to innovation processes and with what effects, and of how this compares with other practices which are considered to be ‘work’, such as care. Analysis must also focus on how others are perhaps more marginalised from these processes and opportunities, and what the consequences of these exclusions might be for the kinds of innovations being developed and patients’ experience of disease and healthcare. It also suggests the need for further consideration of how we might account for and give value to the largely hidden kinds of work that some patients are doing as part of the innovation process.

This leads us to ask whether patients’ emotional management of hope, obligation and entitlement (as well as the emotional ‘dirty work’^60^ 60 Robert McMurray and Jenna Ward, ‘“Why Would You Want to Do That?”: Defining Emotional Dirty Work' (2014) 67 *Human Relations* 1123. of handling despair, alienation and anguish) should be transparently recognised as part of the innovation process. This might mean a different form of accounting for costs and benefits, including financially. Beginning to conceptualise these activities as work has the potential to change how we understand and manage participation in trials or biobanking initiatives, helping us to understand why and how patients might take a stake in the benefits generated by these initiatives. Possible avenues for taking this forward could include participation in priority setting and consideration of different models for benefit sharing or compensation for the work involved in ‘being a patient’. Engaging with these issues about the value of patients’ work and the work of creating value in biomedical innovation is a crucial task for policymakers, sociologists and ethicists in the post-genomics era.

